# Clone temporal centrality measures for incomplete sequences of graph snapshots

**DOI:** 10.1186/s12859-017-1677-x

**Published:** 2017-05-16

**Authors:** Moritz Hanke, Ronja Foraita

**Affiliations:** Leibniz Institute for Prevention Research and Epidemiology - BIPS, Department of Biometry and Data Management, Achterstr. 30, Bremen, Germany

**Keywords:** Dynamic networks, Dynamic graphs, Betweenness, Closeness, Centrality measures, Time varying networks, Shortest temporal path

## Abstract

**Background:**

Different phenomena like the spread of a disease, social interactions or the biological relation between genes can be thought of as dynamic networks. These can be represented as a sequence of static graphs (so called graph snapshots). Based on this graph sequences, classical vertex centrality measures like closeness and betweenness centrality have been extended to quantify the importance of single vertices within a dynamic network. An implicit assumption for the calculation of temporal centrality measures is that the graph sequence contains all information about the network dynamics over time. This assumption is unlikely to be justified in many real world applications due to limited access to fully observed network data. Incompletely observed graph sequences lack important information about duration or existence of edges and may result in biased temporal centrality values.

**Results:**

To account for this incompleteness, we introduce the idea of extending original temporal centrality metrics by cloning graphs of an incomplete graph sequence. Focusing on temporal betweenness centrality as an example, we show for different simulated scenarios of incomplete graph sequences that our approach improves the accuracy of detecting important vertices in dynamic networks compared to the original methods. An age-related gene expression data set from the human brain illustrates the new measures. Additional results for the temporal closeness centrality based on cloned snapshots support our findings. We further introduce a new algorithm called REN to calculate temporal centrality measures. Its computational effort is linear in the number of snapshots and benefits from sparse or very dense dynamic networks.

**Conclusions:**

We suggest to use clone temporal centrality measures in incomplete graph sequences settings. Compared to approaches that do not compensate for incompleteness our approach will improve the detection rate of important vertices. The proposed REN algorithm allows to calculate (clone) temporal centrality measures even for long snapshot sequences.

**Electronic supplementary material:**

The online version of this article (doi:10.1186/s12859-017-1677-x) contains supplementary material, which is available to authorized users.

## Background

Many phenomena can be represented and interpreted as dynamic networks. These consist of vertices and edges that occur and vanish at different time points [1]. Global characteristics of a dynamic network’s topology, e.g. its diameter, may vary over time, but also characteristics of individual vertices, such as their centralities. It is essential to take these dynamics into account when one is interested in crucial vertices and subnetworks characterizing the information flow in dynamic networks and their connectivity. The detection of such vertices or subnetworks is important for different research areas like life, social and computer science to understand empirical phenomena like the spread of a disease in a population, the connectivity within and between peer groups or cyber attacks on computer networks [2–4].

Statistical methods for static networks have been an active and fruitful field for statistical research in the last decades. In recent years the development of probabilistic models for dynamic networks as well as the development of methods for describing key properties of these networks have gained more and more attention [5]. For this purpose, a dynamic network is often represented as a dynamic graph consisting of a vertex set *V* and a temporal edge set *E*. While some authors [5, 6] define a temporal edge as event between two vertices *a* and *b* starting at a particular time point with specific edge duration, others [7–9] define a dynamic network as a sequence of static graphs, so called snapshots, consisting of temporal edge sets *E*_*t*_. The temporal order of the edge set describes the direction of the dynamics. The sequence of snapshots can either consist of static graphs of specific time points, or aggregated static graphs constructed by combining all edges present within a predefined time interval. In many scientific fields, e.g. genetic epidemiology, only static graphs of specific time points are available rather than fully observed dynamic network structures, for example because it is technologically infeasible to determine the exact starting time or duration of an edge between two vertices. Based on the representation of a snapshot sequence it is possible to extend vertex measures like closeness and betweenness centrality from static to dynamic network settings. However, it is inappropriate to apply vertex centrality measures for static settings, to quantify the importance of vertices in a dynamic network because the dynamic topology of the network will be neglected [5, 10]. This is for example the case when a dynamic network is aggregated into a static graph sequence and then ‘classical’ vertex centralities are calculated without taking into account the structural changes within the network over time. Calculating static centrality measures for every vertex of each snapshot and then averaging these values also neglects the the time order of the snapshots. Faisal & Milenkovic correlated static centrality measures with the time of the respective snapshot to calculate centrality values in dynamic networks [11]. However, their approach is not a *temporal* centrality measure because it does not reflect temporal paths. To address this shortcoming we use the concept of temporal paths necessary to appropriately describe the centrality of a vertex in its chronological sequence [12–14].

Tang et al. extended static centrality measures for the use in dynamic networks by accounting for shortest *temporal* paths [8]. Their approach assumes that all network information within a previously chosen window size is aggregated into one snapshot. Kim and Anderson [15] modified the representation of a sequence of graph snapshots into a single directed time graph linking each vertex with its successors in time. Based on this directed time graph the authors slightly reformulated the centrality measures of [8]. Another definition of vertex centrality was given for temporal walks [16] that allow to visit edges multiple times per time point instead of once as with shortest temporal paths. This temporal centrality measure can be interpreted as a temporal version of the static Katz centrality [17].

While the computation of dynamic network characteristics mainly assumes a fully observed dynamic network, there is a lack of approaches for incomplete graph sequences which pose two major challenges:

- An edge in an observed snapshot could have arisen at an earlier and unknown time point in the past and could last until an unknown time point in the future. Hence, starting time and duration of this edge are uncertain.
- Some edges are unobserved because they occur and vanish in the time interval between two consecutive observed snapshots. Such edges are not observed and hence also their influence on the network’s dynamic is difficult to assess.

Both cases will affect temporal centrality measures and are likely to occur in real world applications, e.g. when data of gene expression networks are available only at some – maybe unequally spaced – time points [11, 18] or when rapid changes occur within the network [19]. While some authors propose metrics to quantify the overall stability of the topology of a dynamic network [20–24], the impact on centrality measures due to incomplete information was only investigated for static network settings [25, 26]. The development of temporal centrality measures accounting for incompletely observed dynamic networks is still lacking.

Our work fills this gap by introducing the problem of incomplete graph sequences and proposing an extensions of the temporal betweenness and closeness centralities of Kim & Anderson [15] by using additional snapshots in situations of incomplete graph sequences. These added snapshots are copies of observed snapshots and will be referred to as *clones* in the following. Hence we propose the *clone* temporal betweenness and closeness centrality (CTBC, CTCC). The main purpose of adding clones is to allow more moves along a graph sequence and hence to increase the number of identified temporal paths that could not have been found with the originally observed snapshot sequence. We demonstrate in simulation studies and in an application to a real dynamic gene network that our new approach provides simple improved vertex centrality estimates in situations with incomplete graph sequences. We further considered the computational aspect of our new measures. The time complexity for calculating centrality measures in dynamic graphs depends on the number of vertices and edges as well as on the number of snapshots. Especially, the calculation of temporal centrality measures based on (shortest) temporal paths can be challenging because, unlike static graphs, for dynamic graphs it does not hold that every subpath of a shortest temporal path is again a shortest path. Hence, the search for the shortest temporal path has to visit all relevant subsequences of graphs, i.e. starting from every snapshot up to the last snapshot. Otherwise the full dynamics of the network will not be considered appropriately in the calculated centrality values [15, 27]. To address this time demanding requirement, we propose a novel and easy to implement algorithm called REN (Reversed Evolution Network). Its time complexity is linear in the number of graph snapshots for a fixed number of vertices and edges. This property allows to search for shortest temporal paths in long graph sequences or in a graph sequence that has been augmented by clones. In addition, our simulations suggest that the overall running time of REN benefits from dense and sparse dynamic networks.

## Methods

Let us assume a finite time interval in which a dynamic network has been observed, starting at *t*_*start*_ and ending at *t*_*end*_, where without loss of generality *t*_*start*_=0 and *t*_*end*_=*T*. A dynamic network is represented as a dynamic graph $G^{D}_{0,T}=(V,E_{0,T})$, where we assume a finite set *V* of |*V*| vertices and an edge set *E*_0,*T*_ that can change in the time interval [ 0,*T*]. While we will focus on edge sets *E*_0,*T*_ consisting of temporal *undirected* edges {*a,b*}_*i,j*_∈*E*_0,*T*_ with *a,b*∈*V* that are present in the time interval [ *i,j*] with 0≤*i*<*j*≤*T*, it is straightforward to extend our approach to temporal *directed* edges.

In the following we will present the basic notations to introduce incomplete graph sequences. We will then derive a modified version of the temporal betweenness centrality as an example for our approach using cloned snapshots.

### Graph sequences and shortest temporal paths

To characterize structural properties of a dynamic network a dynamic graph $G^{D}_{0,T}$ is commonly discretized into a time ordered sequence of static graphs $\mathcal {G}=G_{1},G_{2}\ldots, G_{S}$ with corresponding edge sets *E*_*k*_ for *k*∈{1,2,…,*S*}, such that *G*_*k*_=(*V,E*_*k*_). Each edge set *E*_*k*_ of a snapshot *k* consists of all edges that are present in a time window *w*_*k*_ of size *w*≤(*t*_*end*_−*t*_*start*_)=*T*. Thus, the number of snapshots is given by *S*=*T*/*w*.

**Fig. 1:**
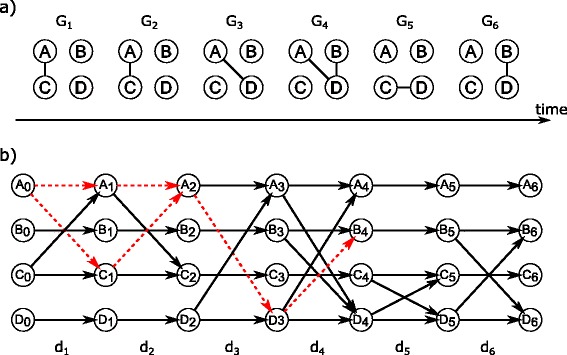
Directed time graph (DTG). A graph sequence $\mathcal {G}$ of snapshots in (**a**) and its representation as a DTG in (**b**). *Horizontal edges* in (**b**) indicate halts on a vertex, *diagonal edges* represent hops. Two shortest temporal paths from vertex *A* to vertex *B* are marked by *red dashed edges*

Sequences of graph snapshots can be represented as directed time graphs (DTG) [15, 21]. Figure 1 shows a graph sequence and its adequate DTG. Each snapshot *G*_*k*_ in Fig. 1 a has a corresponding column *d*_*k*_ of *directed* edges (Fig. 1 b). Hence, every vertex *a*∈*V* of $\mathcal {G}$ occurs *S*+1 times in a DTG, indicated by *a*_0_,*a*_1_,…,*a*_*S*_. The columns *d*_*k*_ of a DTG contain the (undirected) edges of the original snapshot representation plus edges from each vertex to itself at the next time point (horizontal edges). The latter edges represent *halts* in a snapshot; all other edges are called *hops*.

It is possible to formulate an *edge sequence* connecting vertices along the DTG, as indicated by the red dashed edges in Fig. 1 b. We call such sequences *temporal paths*. They consist of a unique combination of hops and halts. The occurrence of an edge is considered by only allowing either one hop or halt per snapshot *k* (or likewise per column *d*_*k*_). Thus, using the representation as a DTG, a temporal path starting at snapshot *k* and ending at snapshot *n* with *k,n*∈{1,2,…,*S*},*k*≤*n* of a graph sequence $\mathcal {G}=G_{1},\ldots,G_{S}$ is defined as an ordered sequence of vertices *p*_*k,n*_(*a,c*)=〈*a*_*k*−1_,…,*c*_*n*_〉 such that *a,c*∈*V*. Note that *p*_*k,n*_(*a,c*) starts with index *k*−1 in a DTG.

Let $\mathbf {P}_{k,n}(a,c)=\bigcup _{m = k}^{n} p_{k,m}(a,c)$, that is the set of all possible temporal paths starting from vertex *a* at snapshot *k* and ending in vertex *c*, at the latest, in snapshot *n*. Note, a temporal path from *a* to *c* can end at *m*≤*n*. If a path path *p*_*k,m*_(*a,c*) exists, the *path length* is defined as |*p*_*k,m*_(*a,c*)|=*m*−*k*+1, which is the number of halts and hops needed to travel from vertex *a* to vertex *c* in the graph sequence *G*_*k*_,…,*G*_*m*_. A *shortest* temporal path *γ*_*k,m,n*_(*a,c*) is then defined as the path *p*_*k,m*_(*a,c*)∈**P**_*k,n*_(*a,c*) with minimum number m, where *c* is reached in snapshot *m*≤*n*. It’s length is |*γ*_*k,m,n*_(*a,c*)|=*m*−*k*+1. The set $\Gamma _{{k,m,n}}(a,c)=\bigcup \gamma _{{k,m,n}}(a,c)$ contains all shortest temporal paths from *a* to *c* within the considered sequence *G*_*k*_,…,*G*_*n*_. Consequently, all shortest temporal paths of *Γ*_*k,m,n*_(*a,c*) have the same path length *m*−*k*+1.

Expanding the above notation, *γ*_*k,m,n*_(*a,b*_*l*_,*c*)∈*Γ*_*k,m,n*_(*a,c*) denotes a shortest temporal path that crosses vertex *b* at snapshot *l*. Therefore, the set $\Gamma _{{k,m,n}}(a,b,c) = \bigcup _{k<l<m} \gamma _{{k,m,n}}(a,b_{l},c) $ contains all shortest paths from *a* to *c* that cross *b* at some snapshot *l*.

If a shortest temporal path *γ*_*k,n,n*_(*a,b*_*l*_,*c*) contains the holds and hops of *p*_*l,n*_(*b,c*) we call *p*_*l,n*_(*b,c*) the *upper temporal subpath* of *γ*_*k,n,n*_(*a,b*_*l*_,*c*). Analogously, if *γ*_*k,n,n*_(*a,b*_*l*_,*c*) contains all edges of *p*_*k,l*_(*a,b*) we call *p*_*k,l*_(*a,b*) a *lower temporal subpath* of *γ*_*k,n,n*_(*a,b*_*l*_,*c*). Additionally, we simply call every sequence of hops and halts of *p*_*k,n*_(*a,c*) starting at a snapshot *l*, *l*>*k*, and ending at a snapshot *m*, *m*<*n*, a temporal subpath of *p*_*k,n*_(*a,c*).

In the following we will show that every upper temporal subpath of a shortest temporal path will always be a shortest temporal path itself even if the lower temporal path is not a shortest temporal path.

#### **Lemma 1**

Given a graph sequence $\mathcal {G}=G_{k},\ldots,G_{l},\ldots, G_{m}, \ldots, G_{n}$, let *γ*_*k,n,n*_(*a,b*_*l*_,*c*) be a shortest temporal path from *a* to *c* that passes vertex *b* at snapshot *l* and ends at snapshot *n*. Then, even if the lower temporal path *p*_*k,l*_(*a,b*) is not a shortest temporal path, the upper temporal path *p*_*l,n*_(*b,c*) is a shortest temporal path, i.e. *p*_*l,n*_(*b,c*)=*γ*_*l,n,n*_(*b,c*).

#### *Proof*

Assume that there exists a temporal path *p*_*l,m*_(*b,c*) from *b* to *c* with |*p*_*l,m*_(*b,c*)|<|*γ*_*l,n,n*_(*b,c*)|. Then it follows that

$$\begin{array}{@{}rcl@{}} |\gamma_{k,n,n}(a,b_{l},c)|&=&|p_{k,l}(a,b)|+|\gamma_{l,n,n}(b,c)| >|p_{k,l}(a,b)|\\&&+|p_{l,m}(b,c)|=|p_{k,m}(a,c)| \end{array} $$

which is contradiction to the assumption that *γ*_*k,n,n*_(*a,b*_*l*_,*c*) is the shortest temporal path from *a* to *c* over *b* at snapshot *l*. □

Note that although *all* subpaths of shortest paths are again shortest path in a *static* directed graph [28], this does not hold for a DTG. As a simple example consider a path *p*_*k*,*n*_(*a*,*c*)=*γ*_*k,n,n*_(*a*,*c*)=*γ*_*k,n,n*_(*a*,*b*_*l*_,*c*)=*γ*_*k,n,n*_(*a,b*_*m*_,*c*), *l*<*m*, from *a* to *c* that passes vertex *b* at snapshots *l* and *m*. Then, |*p*_*k,l*_(*a,b*)|<|*p*_*k,m*_(*a,b*)| and hence *p*_*k,m*_(*a,b*) is not a shortest path although it is a subpath of *γ*_*k,n*_(*a,c*).

While the query for (shortest) temporal paths is only meaningful in graph sequences with at least two snapshots, the *length* of a (shortest) temporal path can be one, if *a* and *c* are connected at the first snapshot of the graph sequence, that is |*p*_*k,n*_(*a,c*)|≥|*γ*_*k,k,n*_(*a,c*)|=1.

### Incomplete graph sequences

**Fig. 2:**
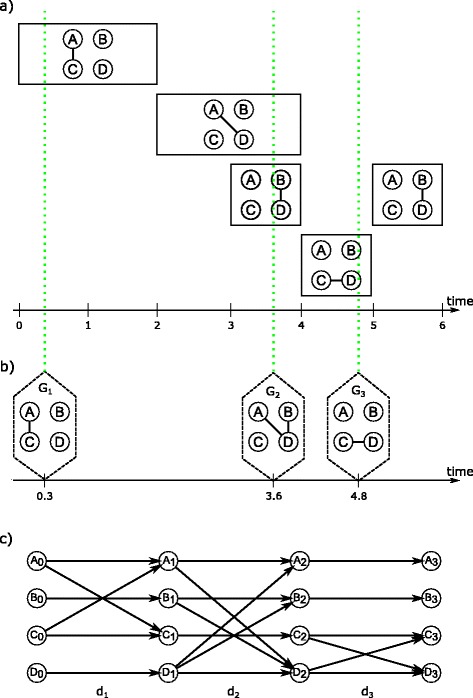
Incomplete graph sequence. An incomplete observed graph sequence in (**b**) and its DTG (**c**) compared to the true but unobserved dynamic network in (**a**). *Solid boxes* in (**a**) represent time intervals of the respective edge occurrence within the true dynamics. *Dashed boxes* in (**b**) indicate snapshots at specific time points and the *green dotted lines* mark the corresponding moments in (**a**). The sequence of graph snapshots yields the incomplete graph sequence

If there is only limited access to *S* snapshots of time points *t*∈ [ 0,*T*], the observed graph sequence $\mathcal {G}$ is incomplete. In this situation it might be impossible to determine exactly when an edge occurs and how long it has existed in the network. Additionally, incomplete sequences might miss edges in total and thus can lead to unobserved edges. Figure 2 gives an example of the impact of incomplete graph sequences. Although in Fig. 2 b the first snapshot *G*_1_ at *t*=0.3 correctly captures the occurrence of edge {*A,C*}, it cannot determine its duration until *t*=2. The true edge sequence of {*A,D*} followed by {*B,D*} cannot be reconstructed because at the next snapshot *G*_2_ (*t*=3.6) both edges are aggregated into one graph. This *masks* their chronological order. Further, the second occurrence of {*B,D*} in the time interval [5,6] is not detected, because the last observation of the dynamic network is *G*_3_ at *t*=4.8, and therefore the edge {*B,D*} is missing in the observed graph sequence. The consequence is that there is no temporal path from *A* to *B* in the observed DTG (Fig. 2 c).

Both, masked edge chronologies and unobserved edges affect the number of observable (shortest) temporal paths in a dynamic network.

### Clone temporal betweenness centrality

In a static network, the betweenness centrality of a vertex *b* measures how easily *b* can be avoided when seeking for shortest paths to get from vertex *a* to *c*, *a*≠*b*≠*c*∈*V*. More precisely, it is the ratio between the number of shortest paths from *a* to *c* passing *b* and the total number of shortest paths from *a* to *c*. This idea has been extended [8, 15] to graph sequences $\mathcal {G}=G_{1},\dots,G_{S}$ consisting of *S* snapshots. Let *σ*_*k,m,S*_(*a,b,c*) denote the cardinality of the set of the shortest paths *Γ*_*k,m,S*_(*a,b,c*) and *σ*_*k,m,S*_(*a,c*) denote the cardinality of *Γ*_*k,m,S*_(*a,c*) for a graph sequence *G*_*k*_,…,*G*_*S*_. The *temporal betweenness centrality* (TBC) of vertex *b* is then defined as:

1 $$\begin{array}{@{}rcl@{}}  TBC_{1,S}(b)=\sum_{k=1}^{S-1} \sum_{\substack{a,c \in V\backslash b \\ \sigma_{k,m,S}(a,c) >0}} \frac{\sigma_{k,m,S}(a,b,c)}{\sigma_{k,m,S}(a,c)}. \end{array} $$

The second sum in Eq. (1) accounts for all shortest paths starting from vertex *a* and the first sum ensures that all subsequences starting at a snapshot after *k*, *G*_*l*_,…,*G*_*S*_, *l*>*k*, are included in the calculation of this measure. This is necessary to adequately capture the complete dynamic behaviour in the network over time [27]. For example, consider a graph sequence with all vertices connected to each other at the first snapshot but with fewer connections at the following snapshots. Applying the TBC without summing over all later subsequences will not represent the dynamics after the first snapshots because all shortest temporal paths will be of length one due to the fully connected first snapshot. However, TBC cannot explicitly handle incomplete graph sequences and hence it will miss (shortest) temporal paths when calculating a vertex’ centrality.

Consider Fig. 2 and assume that we have only observe the sequence as shown in Fig. 2 b; what can then be inferred about the true underlying sequence in Fig. 2 a? It is obvious that the edge {*A,C*} in snapshot *G*_1_ must have occurred before the next observed snapshot *G*_2_. The edges {*A,D*} and {*B,D*} observed in snapshot *G*_2_ on the contrary must have occurred in the dynamic network at a time point between snapshots *G*_1_ and *G*_2_ but we do not know the order of occurrence and thus the possible temporal paths. Our proposal is to *fill* the gap between snapshots with additional snapshots, in order to reveal additional (shortest) temporal paths that are likely to exist. These added snapshots are copies of observed snapshots and will be referred to as *clones*.

#### **Definition 1**

Given a static graph *G*_*k*_(*V,E*_*k*_) of snapshot *k* we define clones of *G*_*k*_ as $G_{k,j_{k}}(V,E_{k,j_{k}})$ such that $G_{k,j_{k}}(V,E_{k,j_{k}}) = G_{k}(V, E_{k})$ for *j*_*k*_=1,2,…,*J*_*k*_.

Based on definition 1 and using the notation $G_{k,j_{k}}$ for $G_{k,j_{k}}(V,E_{k,j_{k}})$ we can now define a cloned graph sequence.

#### **Definition 2**

Given a original graph sequence *G*_1_,*G*_2_,…,*G*_*S*_ and clones $G_{k,j_{k}}$ with *k*=1,2,…,*S* and *j*_*k*_=1,2,…,*J*_*k*_ a cloned graph sequence is defined as the ordered sequence $\phantom {\dot {i}\!}G_{1,1},G_{1,2},\ldots,G_{k,j_{k}},\ldots, G_{S,J_{S}}$.

Augmenting the original graph sequence with clones $G_{k,j_{k}}$ raises the question of how to choose the number of clones *J*_*k*_ per snapshot. This is generally flexible and may vary depending on the application. We propose the following three plausible approaches:

1. Adding a sufficient number of clones *J*_*k*_ per snapshots *k* such that any *static* path in *G*_*k*−1_∪*G*_*k*_ not presented in *G*_*k*−1_ and *G*_*k*_ alone can be found as a *temporal* path. This is always possible and depends on the number of different edges between *G*_*k*−1_ and *G*_*k*_.
2. Adding clones based on assumptions about the expected duration of the occurrence of edges.
3. If the number of unobserved discrete time points between *G*_*k*−1_ and *G*_*k*_ is known a corresponding number of clones can be added.

**Fig. 3:**
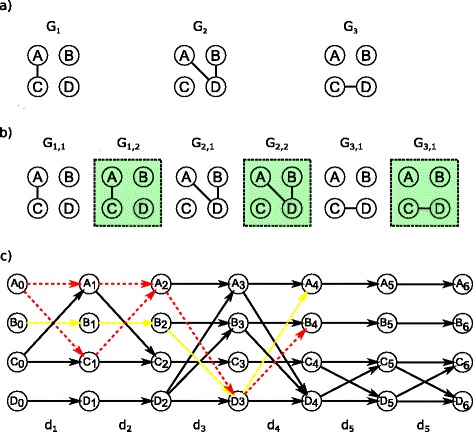
Cloned graph sequence. The incomplete observed graph sequence in (**a**) is based on the incomplete graph sequence of Fig. 2. The first two observed snapshots are cloned as shown in the graph sequence in (**b**) and the respective DTG in (**c**). *Green boxes* indicate clones. Both true temporal paths from *A* to *B* (*red dashed arrows*) in the original complete graph sequence were found due to cloning (see Fig. 1). However, a spurious (shortest) temporal path was also detected that is not present in the original sequence (indicated by the *yellow dashed arrows*)

Figure 3 shows an example of temporal path search in a graph sequence including cloned snapshots. Given the true dynamic network depicted in Fig. 1 and the observed snapshots of Fig. 2 b, we constructed the graph sequence presented in Fig. 3 a and decided to clone each of its snapshot once, resulting in the graph sequence of Fig. 3 b. As shown in Fig. 3 c, clones can detect shortest temporal paths that are in fact a true shortest temporal paths (red dashed arrows). However, cloning compensates only for unobserved edge durations and ordering of occurrences, but it cannot detect unobserved edges and hence also no temporal paths that contain these unobserved edges. Furthermore, if cloning overestimates edge durations or the order of occurrence (as for the edge (*B,D*)), it might detect false shortest temporal paths (indicated by the yellow dashed arrows). We call this problem *excess of cloning* and discuss its implications in more detail in the simulation section.

Exploiting the idea of cloning snapshots, we extend the TBC of Eq. (1) to a *clone temporal betweenness centrality* (CTBC):

2 $$\begin{array}{@{}rcl@{}}  CTBC_{1,S}(b)= \sum_{k=1}^{S} \sum_{j_{k}=1}^{J_{k}} \sum_{\substack{a,c \in V\backslash b \\ \sigma_{k, m,S}^{j_{k}}(a,c) >0}} \frac{\sigma^{j_{k}}_{k,m,S}(a,b,c)}{\sigma^{j_{k}}_{k,m,S}(a,c)}, \end{array} $$

where $\sigma _{k,m,S}^{j_{k}}(a,b,c)$ denotes the number of shortest temporal paths from *a* to *c* passing *b*, starting at the *j*_*k*_-th clone of snapshot *k*. Similarly, $\sigma ^{j_{k}}_{k,m,S}(a,c)$ denotes the total number of shortest paths from *a* to *b* starting at the *j*_*k*_-th clone of snapshot *k*. The CTBC successively sums the sequence of observed and cloned snapshots starting at the *j*_*k*_-th clone of snapshot *k* until the last clone of snapshot *S*. CTBC is applicable for graph sequences of directed and undirected temporal networks. The idea of cloning snapshots when calculating temporal centrality measures can also easily be applied to other temporal centrality measures like the temporal closeness centrality (see Additional file 1).

### REN: a new algorithm for finding shortest temporal paths

An appropriate algorithm is necessary to calculate the above temporal centrality measures. The summation over all subsequences in Eqs. 1 and 2 can be computationally demanding for long graph sequences because a shortest temporal path in *G*_*k*_,…,*G*_*S*_ might not be a (shortest) temporal path in *G*_*k*+1_,…,*G*_*S*_ which necessitates a new query. As a consequence, a new search for shortest temporal paths has to be started for each snapshot of the graph sequence *G*_*k*_,…,*G*_*S*_. For example, there are two shortest temporal paths starting from vertex *A* at snapshot 1 and ending at vertex *B* at snapshot 4 in Fig. 1. Both paths have to pass vertex *D* at snapshot 3, meaning that a temporal path starting at snapshot 4 or later cannot be subpath of these shortest temporal paths.

Our REN algorithm tackles the problem of consecutive queries by searching for temporal paths in the reversed order of snapshots, defined as $\mathcal {G}^{*}=G_{S},\ldots,G_{1}$. A *reversed temporal path* is defined as $p^{*}_{n,k}(c,a)=\langle c_{n}, \ldots, a_{k-1} \rangle =\texttt {rev}\left (p_{k,n}(a,c)\right)$, where rev(·) is the function that reverses the edge directions in a DTG and therefore the order of the vertices of a temporal path. The basic idea is then to move along all reversed temporal paths starting from a specific vertex *c* at snapshot *S* until snapshot 1 and to store each descendent vertex *b* of *c* and its lowest snapshot number *k* where *b* is connected to *c* by an edge or temporal path. Even if there are shortest temporal paths found before reaching the first snapshot it is crucial to move along all reversed temporal paths up to the first snapshot of the considered graph sequence. Otherwise shortest temporal paths that start at or near the first snapshot are not found.

In the following, we will prove that the computational time of REN is linear with respect to the number of snapshots *S* when searching for all shortest temporal paths in *G*_*k*_,…,*G*_*S*_, ∀ *k*∈ [ 1,*S*−1]. First, we prove that a query along a particular reversed shortest temporal path finds all upper temporal subpaths that are also shortest temporal paths too.

#### **Lemma 2**

Let *G*_*k*_,…,*G*_*n*_,*k*<*n*, be a graph sequence and let *γ* *k,n,n*′(*a,c*)=*p* *k,n*′(*a,c*) be a specific shortest temporal path in *Γ*_*k,n,n*_(*a,c*). Then, moving along the reversed temporal path $p_{n,k}^{*}(c,a)=\text {\texttt {rev}}\left (p'_{k,n}(a,c)\right)$ from vertex *c* to vertex *a* finds all *n*−*k* shortest temporal paths *γ* *l,n,n*′(*b,c*), *k*≤*l*<*n* from any vertex *b* to vertex *c* that are upper temporal subpaths of *γ* *k,n,n*′(*a,c*)=*γ* *k,n,n*′(*a,b*_*l*_,*c*) and for which *b*=*b*_*l*_∈*γ* *k,n,n*′(*a,b*_*l*_,*c*).

#### *Proof*

A specific shortest temporal path *γ* *k,n,n*′(*a,c*)∈*Γ*_*k,n,n*_(*a,c*) is characterised by a unique combination of *n*−*k* hops and halts. This temporal path contains then *n*−*k* upper temporal subpaths, each starting at a different snapshot *k,k*+1,…,*n*−1. For *l*=*k* it directly follows that *γ* *l,n,n,*′(*a,c*)=*γ* *k,n,n,*′(*a,c*).Now, let *l*=*k*+1 and let *b*∈*V*∖*c* be a vertex on *γ* *k,n,n,*′(*a,c*), that is it holds *γ* *k,n,n,*′(*a,b*_*l*_,*c*)=*γ* *k,n,n*′(*a,c*). Applying Lemma 1 yields that the upper temporal subpath $p^{\prime }_{l,n}(b,c)$ of $p^{\prime }_{k,n}(a,c)=\gamma '_{{k,n,n}}(a,b_{l},c)$ is also a shortest temporal path *γ* *l,n,n*′(*b,c*). This holds for all further *l*=*k*+2,…,*n*−1, i.e. *γ* *k,n,n*′(*a,c*) contains *n*−*k* upper temporal subpaths (including *γ* *k,n,n*′(*a,c*) itself) that are shortest temporal paths.Then, it follows that $p^{*}_{n,k}(c,a)=\text {\texttt {rev}}\left (p'_{k,n}(a,c) \right)$ contains all reversed upper temporal subpaths $p^{*}_{n,l}(c,a)=\text {\texttt {rev}}\left (p'_{l,n}(a,c) \right)=\text {\texttt {rev}}\left (\gamma '_{{l,n,n}}(a,c) \right)$ with *k*≤*l*<*n*. Thus, following the reversed upper temporal path $p^{*}_{n,k}(c,a)$ reveals all *n*−*k* shortest temporal paths of *γ* *k,n,n*′(*a,c*). □

With Lemma 2 it is possible to show that one query for all reversed temporal paths starting at vertex *c* is sufficient to reveal all shortest temporal paths that end at *c* of a graph subsequence starting at a snapshot at or after *k*.

#### **Theorem 1**

Let $\mathcal {G}=G_{k},\ldots, G_{n}, k<n,$ be a graph sequence and let $\boldsymbol {\Gamma }_{k,n}(\cdot, c)=\bigcup _{l=k}^{n-1} \bigcup _{m = l}^{n} \bigcup _{a \in V\backslash c} \gamma _{{l,m,n}}(a,c)$ be the set of all shortest temporal paths that start from any vertex at snapshot *l*≥*k* and end in vertex *c* at snapshot *m*≤*n*. Further, let $\mathbf {p}^{*}_{n,k}(c,\cdot)=\bigcup _{a \in V\backslash c} p^{*}_{n,k}(c,a)$ be the set of all reversed temporal paths starting from vertex *c* at snapshot *n* and ending at any vertex *a*∈*V*∖*c* at snapshot *k*. Then, every shortest temporal path *γ*_*l,m,n*_(*a,c*)∈***Γ***_*k,n*_(·,*c*) is a reversed subpath of a reversed temporal path in $\mathbf {p}^{*}_{n,k}(c,\cdot)$ and is therefore obtained by moving along every $p^{*}_{n,k}(c,a) \in \mathbf {p}^{*}_{n,k}(c,\cdot)$.

#### *Proof*

Every shortest temporal path *γ*_*l,m,n*_(*a,c*)∈***Γ***_*k,n*_(·,*c*) is a subpath of a temporal path in $\mathbf {p}_{k,n}(\cdot,c)=\bigcup _{a \in V \backslash c} p_{k,n}(a,c) $. Then, the set of all reversed temporal paths $\mathbf {p}^{*}_{n,k}(c,\cdot)=\text {\texttt {rev}}\left (\mathbf {p}_{k,n}(\cdot,c) \right)$ also includes the set of reversed shortest temporal paths $\boldsymbol {\Gamma }^{*}_{k,n}(\cdot, c)=\text {\texttt {rev}}\left (\boldsymbol {\Gamma }_{k,n}(\cdot, c) \right)$.

Lemma 2 shows for every specific shortest temporal path *γ* *l,m,m*′(*a,c*)∈***Γ***_*k,n*_(·,*c*) that the reversed path $p^{*}_{m,l}(c,a)=\texttt {rev}\left (p'_{l,m}(a,c) \right)=\texttt {rev}\left (\gamma '_{{l,m,m}}(a,c) \right)$ contains all *m*−*l* upper subpaths of *γ* *l,m,m*′(*a,c*) that are also shortest temporal paths. Finally, because $p^{*}_{m,l}(c,a)$ is a subpath of $p^{*}_{n,k}(c,a) \in \mathbf {p}^{*}_{n,k}(c,\cdot)$, it will be detected by moving along the reversed temporal paths of $\mathbf {p}^{*}_{n,k}(c,\cdot)$. This holds for all *a*∈*V*. □

Let $\boldsymbol {\mathcal {P}}_{1,S}(\cdot,c)=\bigcup _{k=1}^{S} \bigcup _{m = k}^{S} \bigcup _{b\in V\backslash c} p_{k,m}(b,c)$ denote the set of all temporal paths starting from any vertex *b*∈*V*∖*c* at a snapshot *k* and ending in vertex *c* not later than at snapshot *S*. The set of all reversed temporal paths starting in vertex *c* and ending in any vertex *b*≠*c* is $\boldsymbol {\mathcal {P}}^{*}_{S,1}(c,\cdot)=\text {\texttt {rev}}\left (\boldsymbol {\mathcal {P}}_{1,S}(\cdot,c) \right)$. Further, let **N**_*k*_(*c*)⊆*V*∖*c* be the set of all neighbours of *c*, i.e. adjacent vertices of *c*, at snapshot *k*. By applying Theorem 1 to all *c*∈*V*, REN can be outlined as follows:

1. Reverse the order of the observed snapshot sequence as $\mathcal {G}^{*}=G_{S},\ldots,G_{1}$.
2. Select a start vertex *c* and set $\boldsymbol {\mathcal {P}}^{*}_{S,1}(c,\cdot)=\emptyset $.
3. For snapshot *k*=*S*: Find all adjacent vertices *b*∈**N**_*S*_(*c*). Each edge between *c* and *b* forms a reversed temporal path $p^{*}_{S,S}(c,b)=\langle c_{S},b_{S-1} \rangle $ and is stored in the set $\boldsymbol {\mathcal {P}}^{*}_{S,1}(c,\cdot)$.
4. For snapshots *k*=*S*−1,…,1:
  - List all adjacent vertices *b*∈**N**_*k*_(*c*). Each edge between *c* and *b*∈**N**_*k*_(*c*) forms a reversed temporal path $p^{*}_{k,k}(c,b)=\langle c_{k},b_{k-1} \rangle $ and is stored in the set $\boldsymbol {\mathcal {P}}^{*}_{S,1}(c,\cdot)$. Set *p*_*k,k*_(*b,c*)=〈*b*_*k*−1_,*c*_*k*_〉=*γ*_*k,k,S*_(*b,c*).
  - List all vertices *a*∈*V*∖{**N**_*k*_(*c*)∪*c*} that are adjacent to any vertex *b* for which $p^{*}_{m,k+1}(c,b) \in \boldsymbol {\mathcal {P}}^{*}_{S,1}(c,\cdot)$.
    Join the reversed temporal paths $p^{*}_{k+1,k}(b,a)$ and $p^{*}_{m,k+1}(c,b)$ at vertex b to obtain the reversed temporal path $p^{*}_{m,k}(c,a)$ and store it in $\boldsymbol {\mathcal {P}}^{*}_{S,1}(c,\cdot)$.
    Set $\gamma _{k,m,S}(a,c)=p_{k,m_{\min }}(a,c)$ for $m_{\min }=\arg \min _{\substack {m: k<m \leq S }}|p^{*}_{m,k}(c,a)|$.
5. Repeat steps 2 up to 4 for all other *c*∈*V*.

**Fig. 4:**
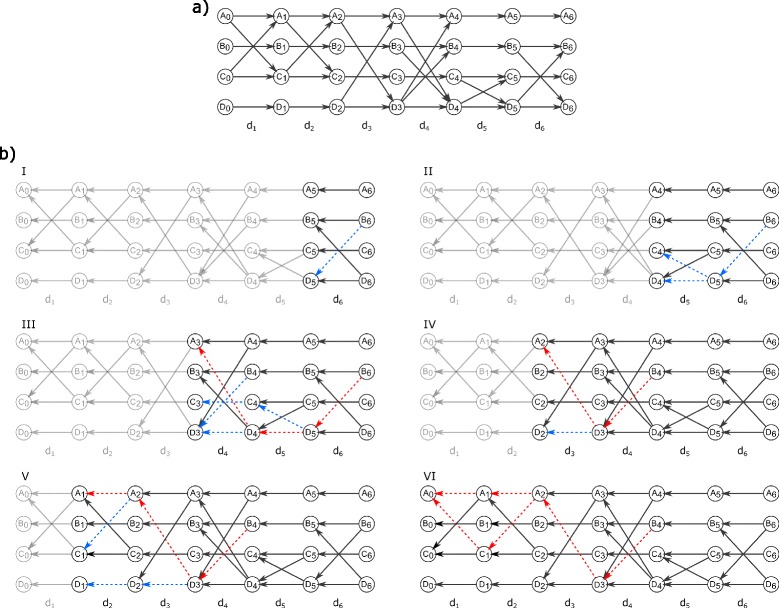
Example for the algorithm procedure of REN. A DTG representation of a graph sequence $\mathcal {G}=G_{1},\ldots,G_{6}$ in (**a**). The six reversed DTGs in (**b**) demonstrate the stepwise query for shortest temporal paths *γ*_1,*l*,6_(*A,B*) from *A* to *B* in a successively reversed graph sequence. *Blue dashed edges* indicate hops and halts that potentially are parts of shortest temporal paths. *Red dashed edges* indicate detected shortest temporal paths starting at a specific snapshot *l*

Figure 4 b gives an example of how REN finds all shortest temporal paths starting at vertex *A* and ending at vertex *B* in at least one relevant subsequence of graphs. This is achieved by applying one query for shortest temporal paths only once over the reversed snapshot order for each vertex. The graph sequence is represented as a DTG (Fig. 4 a) and without loss of generality we focus on the shortest temporal paths starting from *A*_0_ and ending at *B*_6_ and show that the algorithm finds all shortest temporal paths for which *B* is a destination vertex by one linear query. The blue dashed edges indicate steps on a reversed temporal path which are potential edges of a shortest temporal path. Red dashed edges indicate shortest temporal paths whenever it was detected by following a reversed temporal path. We start the query from vertex *B*_6_ at snapshot *S*=6 and follow all its reversed temporal paths up to snapshot 1 (cf. images in Fig. 4 b I to VI). In column *d*_6_ in image I, vertex *B* has two adjacent vertices, forming the hob 〈*B*_6_,*D*_5_〉 and the halt 〈*B*_6_,*B*_5_〉. Focusing on *D*_5_, there is one hop 〈*D*_5_,*C*_4_〉 and one halt 〈*D*_5_,*D*_4_〉 in *d*_5_ from snapshot 5 to 4. The first reversed temporal path that connects *B* and *A* is detected in *d*_4_ via 〈*D*_4_,*A*_3_〉 and according to Lemma 1 it reveals the shortest temporal path *γ*_4,6,6_(*A,B*)=〈*A*_3_,*D*_4_,*D*_5_,*B*_6_〉. The paths $p^{*}_{4,4}(B,D)=\langle B_{4}, D_{3} \rangle $ in *d*_4_ and $p^{*}_{3,3}(D,A)=\langle D_{3},A_{2} \rangle $ in *d*_3_ add up to the shortest temporal path *γ*_3,4,6_(*A,B*)=〈*A*_2_,*D*_3_,*B*_4_〉 in Fig. 4 b IV. Hence, we can infer that any temporal paths from *A* to *B* starting before snapshot 4 and ending after snapshot 4, are shortest temporal paths. The detection of *γ*_3,4,6_(*A,B*) has the following implications: further shortest paths starting at a snapshot *l*<4 must end in *B*_4_ or before. This means also that stored edges beyond snapshot 4 like 〈*C*_4_,*D*_5_〉 or 〈*D*_4_,*D*_5_〉 cannot be any longer part of further shortest temporal paths. Since 〈*D*_4_,*D*_5_〉 is part of *γ*_4,6,6_(*A,B*), we concluded that *γ*_4,6,6_(*A,B*) is not part of a shortest temporal path starting at a snapshot *l*<4. Figure 4 b V and VI complete the query for shortest temporal paths via reversed temporal paths.

**Fig. 5:**
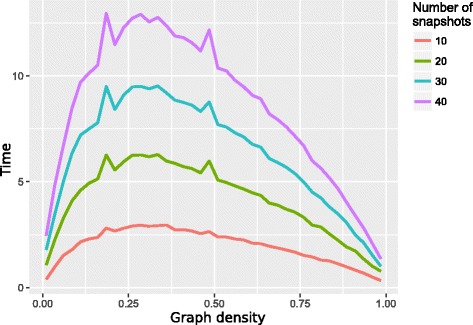
Computing time of REN. Average time (in seconds) needed to calculate the CTBC for 200 vertices using the REN algorithm. *Colours* indicate graph sequences with different number of snapshots. Each snapshot is based on a random Erdős-Rényi model with graph densities ranging from 0.03 up to 0.96. Calculations were done on a single core of a 2.53GHz Intel hexacore processor using 8GB memory

Usually, the time complexity of algorithms for calculating temporal centrality measures based on shortest temporal paths are dominated by the number of snapshots. The authors of the original version of TBC indicate that the time complexity of their algorithm is cubic in the number of snapshots [15]. For long graph sequences they therefore propose to use larger window sizes *w* to discretize the dynamic graph $G^{D}_{0,T}$ into a reduced number of snapshots. This obviously results in a loss of information affecting the accuracy of the temporal centrality measures. REN improves on this limitation because it requires for each vertex only one search over $\mathcal {G}^{*}$. Thus its time complexity is linear in the number of snapshots. Hence, the calculation of centrality measures like TBC becomes feasible in settings with long graph sequences but also for graph sequences with additional snapshots like the proposed CTBC. For example, TBC and CTBC have an overall running time of $\mathcal {O}(S\cdot |V|^{3})$ using REN. REN’s running time benefits from *sparse* and *dense* graphs (see Fig. 5). While the first is obvious due to the small number of temporal paths, the latter can be explained by step 4 of our algorithm. In a dense graph sequence, the number of edges is close to the maximum number of edges for every snapshot, that is most vertices *b* will be *b*∈**N**_*k*_(*c*). Thus, the expensive search of step 4(*b*) can be omitted for these vertices.

If the observed graph sequence is represented as edge list for each snapshot, the space complexity of our algorithm is $\mathcal {O}(S \cdot |E_{(1,S)}| + S \cdot |V|^{2})$, where $|E_{(1,S)}|=\sum _{k=1}^{S}|E_{k}|$ denotes the total number of edges in the dynamic network. The second term denotes the space needed to save all temporal paths of the graph sequence. Note, in the worst case scenario, i.e. when each snapshot contains a saturated graph, space complexity will be $\mathcal {O}(2\cdot S \cdot |V|^{2})$.

## Results

### Simulation study

We define a *group infection network* (GIN) to compare the performance of the CTBC to the TBC in an incomplete graph sequence setting. A GIN contains $M \in \mathbb {N}$ subgraphs *G*(*V* ^(*m*)^,*E* ^(*m*)^),*m*=1,…,*M*. GINs are either undirected or directed, but neither multiple edges between vertices nor loops (i.e. {*a,a*}) are allowed. The probability *p* of an edge is the sum of a baseline probability *τ* and the probability *τ*_+_=*D* ^(*m*)^(*a*)/|*E* ^(*m*)^|, where *D* ^(*m*)^(*a*) denotes the degree of a node *a*∈*V* ^(*m*)^ (i.e. the number of its incident edges) and |*E* ^(*m*)^| denotes the total number of edges in subgraph *m*. Thus, *τ*_+_ reflects a rich-get-richer principle.

We used the representation of a graph sequence consisting of *k*=1,…,*S* snapshots to simulate a GIN as a dynamic network. The initial GIN contains no edges. At snapshot *k*=1 a first vertex is randomly chosen and edges connecting it with any other vertices generated independently with probability *p*. At snapshots *k*≥2 all vertices having one or more incident edges are allowed to connect with other vertices of the same subgraph with probability *p*. After *κ*·*m*<*S* snapshots, $\kappa \in \mathbb {N}$, a connected vertex is randomly chosen as bridge vertex *b*. At the next snapshot, only the bridge vertex builds an edge with a vertex from the next subgraph *m*+1, meaning that only *b* has neighbours in *V* ^(*m*)^ and *V* ^(*m*+1)^. This process is repeated until *k*=*S*. Edges within a GIN remain for $\lambda \in \mathbb {N}$ snapshots and will then vanish. The dynamic of a GIN depends on *λ*, where small values of *λ* lead to rapid changes in the network structure whereas high values of *λ* yield slow changes in the dynamic structure. A dynamic GIN $\mathcal {G}(V,M,S,\tau, \tau _{+},\kappa, \lambda)$ is thus defined by seven parameters.

We generated GINs containing 10 subgraphs, each consisting of 5,10,20,40 or 80 vertices, given an overall network size of |*V*|∈[50,100,200,400,800], respectively. The GIN parameters were set to *τ*=0.0125, *κ*=8 and *λ*=1,2,…,10, i.e. edge durations ranged from 1% to 10% of the total number of snapshots. We simulated 500 undirected GINs for each combination of parameters and, based on the *complete* graph sequence of 100 snapshots, we calculated the TBC from Eq. 1 for each vertex. Vertices were ranked according to their TBC values to make them comparable across graph sequences with different number of snapshots. Ranks of vertices with the same centrality value were averaged. The ranks of the *true* TBC is our reference in the following comparison.

Of each simulated complete graph sequence the *incomplete* graph sequences were generated by randomly drawing *α*=10*%*,20*%*,…,50*%* snapshot, i.e. containing 10, 20, 30, 40 and 50 snapshots. TBC and CTBC (cf. Eq. 2) were estimated for each vertex and ranks were assigned according to their respective centrality values. To calculate CTBC, we set the number of clones equal to the number of unobserved snapshots between two observed snapshots, following our third proposed approach regarding the question how to choose the number of clones. This implies a tendency to overestimate the edge duration. As a consequence false temporal paths might be included (see next section).

For every simulation run, Spearman’s rank correlation coefficient *ρ* between the ranks based on the true TBC values and the TBC respectively CTBC values of the incomplete graph sequence were computed. A high positive *ρ* indicates that the centrality measure relying on incomplete information ranks the vertices similar to the true ranks. In addition, the detection rate was assessed, which is the proportion of how often the most important vertex (rank 1) in the incomplete graph sequences matches the true most important vertex of the complete graph sequence in all simulation runs.

**Fig. 6:**
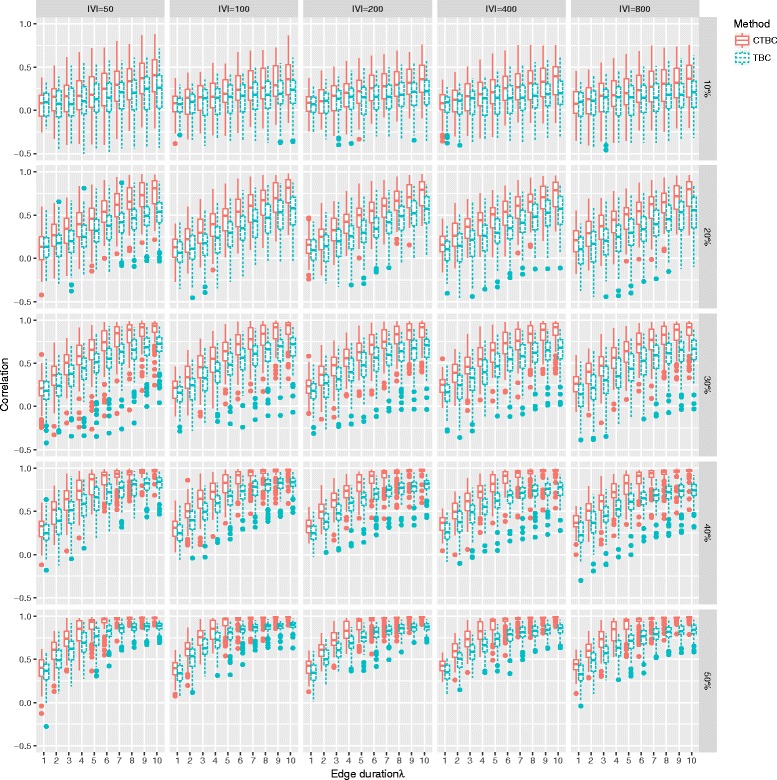
Spearman’s rank correlation coefficient *ρ* for TBC and CTBC in an undirected GIN scenario. Box plots of *ρ* for TBC (*dotted*) and CTBC (*solid*) based on different combinations of number of vertices (|*V*|∈[50,100,200,400,800]), different proportions of randomly observed snapshots (0.2, 0.3, 0.4, 0.5) of the original graph sequence consisting of 100 snapshots and different edge durations (*λ*∈[1,2,…,10]). The results are based on 500 simulation runs for every combination

The Box plots in Fig. 6 show the results of the rank correlation *ρ* for different edge durations *λ*, observation rate *α* and network sizes |*V*|. In all incompleteness scenarios, CTBC outperforms TBC for all *λ* except for the combination (*α*=10*%*, *λ*=1). This could be due to an excess of cloning. As expected, longer edge durations (*λ*>1) considerably improve the performance of CTBC compared to TBC. In addition, the results indicate that the improvement is independent of the network size |*V*| (columns of Fig. 6). Interestingly, CTBC was strongly correlated (*ρ*≈1) with the true TBC for longer edge durations in settings where at least 40% snapshots were observed, while TBC reached a plateau at a lower correlation. In general, if only *α*=10*%* of all snapshots were observed, both methods were weakly correlated with the true TBC, even in situations with an edge duration of *λ*=10 indicating that long edge durations cannot compensate for missing edge observations.

**Fig. 7:**
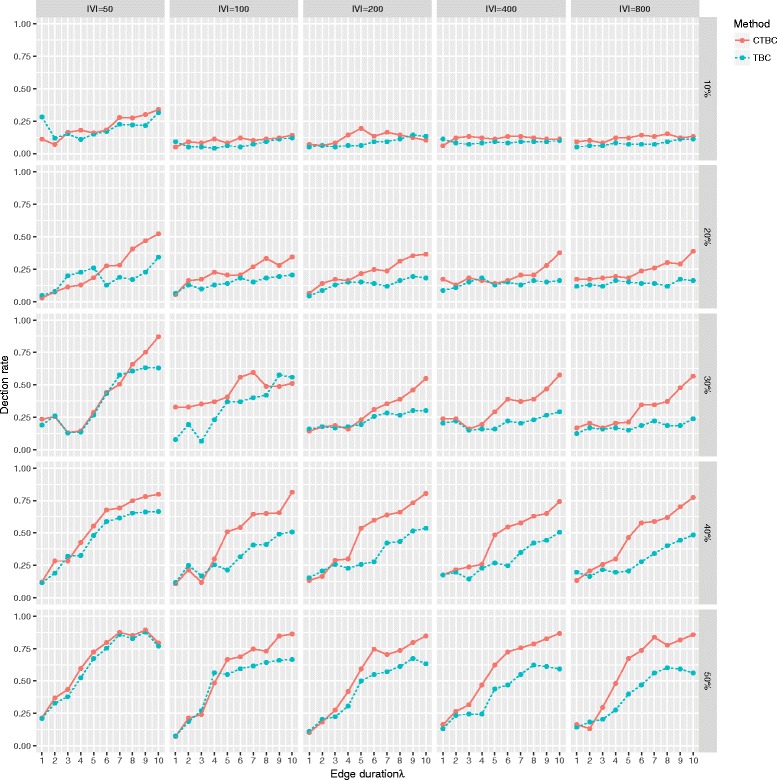
Detection rate of the most important vertex based on TBC and CTBC in an undirected GIN scenario. Detection rate for TBC (*dotted*) and CTBC (*solid*) based on different combinations of number of vertices (|*V*|∈[50,100,200,400,800]), different proportions of randomly observed snapshots (0.2, 0.3, 0.4, 0.5) of the original graph sequence consisting of 100 snapshots and different edge durations (*λ*∈[1,2,…,10]). The results are based on 500 simulation runs for every combination

Figure 7 shows the detection rate for the most important vertex. While TBC and CTBC had poor detection rates in settings of low observation rates (*α*=10*%*), the detection rate of CTBC tended to be better in settings with larger observation rates, especially in combination with longer edge durations.

**Fig. 8:**
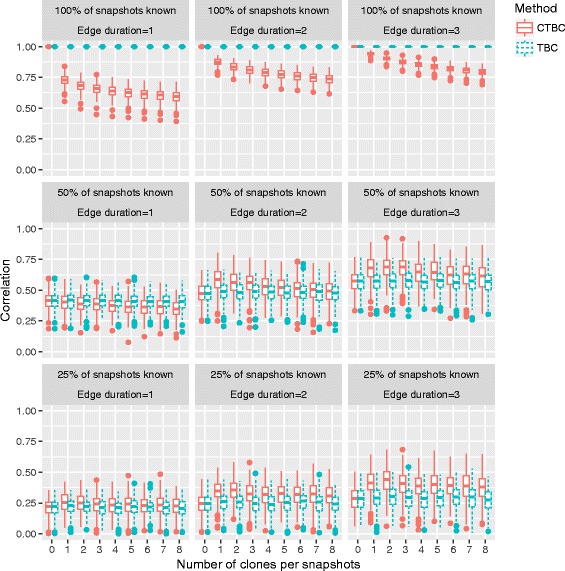
Spearman’s rank correlation coefficient *ρ* for TCC and CTCC in an undirected GIN scenario. Box plots of *ρ* for TCC (*dotted*) and CTCC (*solid*) based on different combinations of number of vertices (|*V*|∈[200,400,800]), different proportions of randomly observed snapshots (0.2, 0.3, 0.4, 0.5) of the original graph sequence consisting of 100 snapshots and different edge durations (*λ*∈[1,2,…,10]). The results are based on 500 simulation runs for every combination

**Fig. 9:**
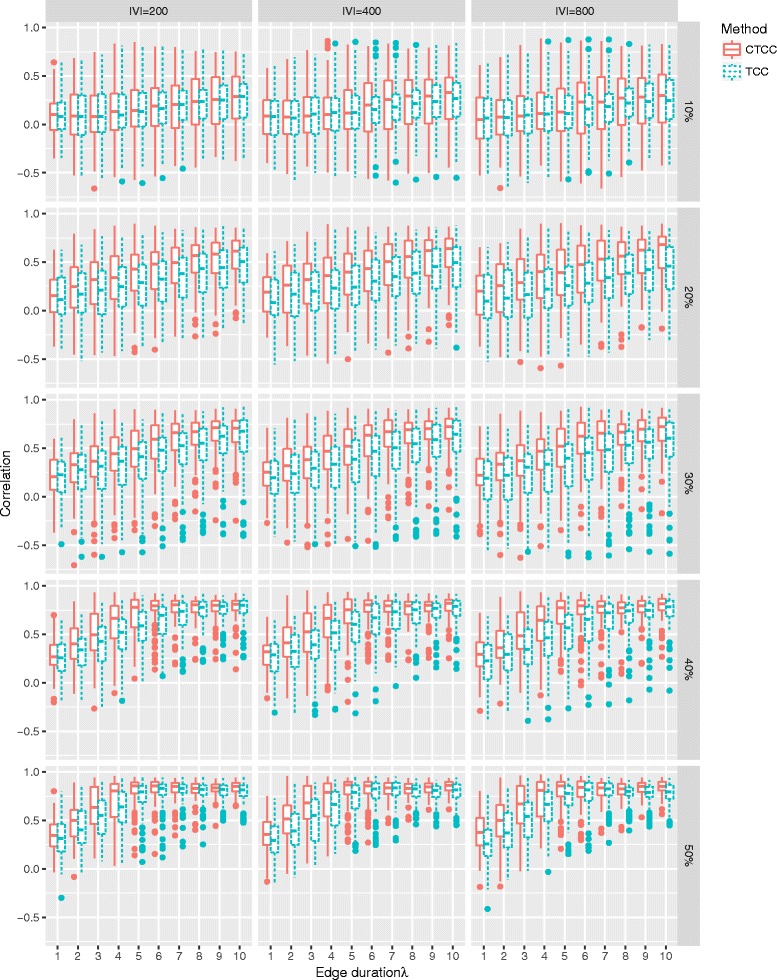
Detection rate of the most important vertex based on TCC and CTCC in an undirected GIN scenario. Detection rate for TCC (*dotted*) and CTCC (*solid*) based on different combinations of number of vertices (|*V*|∈[200,400,800]), different proportions of randomly observed snapshots (0.2, 0.3, 0.4, 0.5) of the original graph sequence consisting of 100 snapshots and different edge durations (*λ*∈[1,2,…,10]). The results are based on 500 simulation runs for every combination

The simulation results for the temporal closeness centrality support our proposal of cloning snapshots, even if the benefit was smaller than for the temporal betweenness centrality, especially regarding the detection rate of the most important vertex (see Fig. 8 and Fig. 9).

### Excess of cloning

As mentioned before, an excess of cloning can introduce false (shortest) temporal paths which lead to biased centrality values. In a further simulation study, we evaluated this bias by generating a GIN with the given parameters |*V*|=200, *M*=10, *τ*=0.0125, *κ*=8, *S*=50 and *λ*=1,2,3. Incomplete graph sequences were sampled assuming an observation rate of *α*=25*%*,50*%*,100*%*. That means, for example in the scenario *α*=100*%* all true snapshots were observed and for each snapshot a specified number of clones were wrongly introduced. As before, true ranks were based on the TBC values for the original graph sequence. For the calculation of CTBC, we fixed the number of clones to *n*_*c*_=0,…,8.

**Fig. 10:**
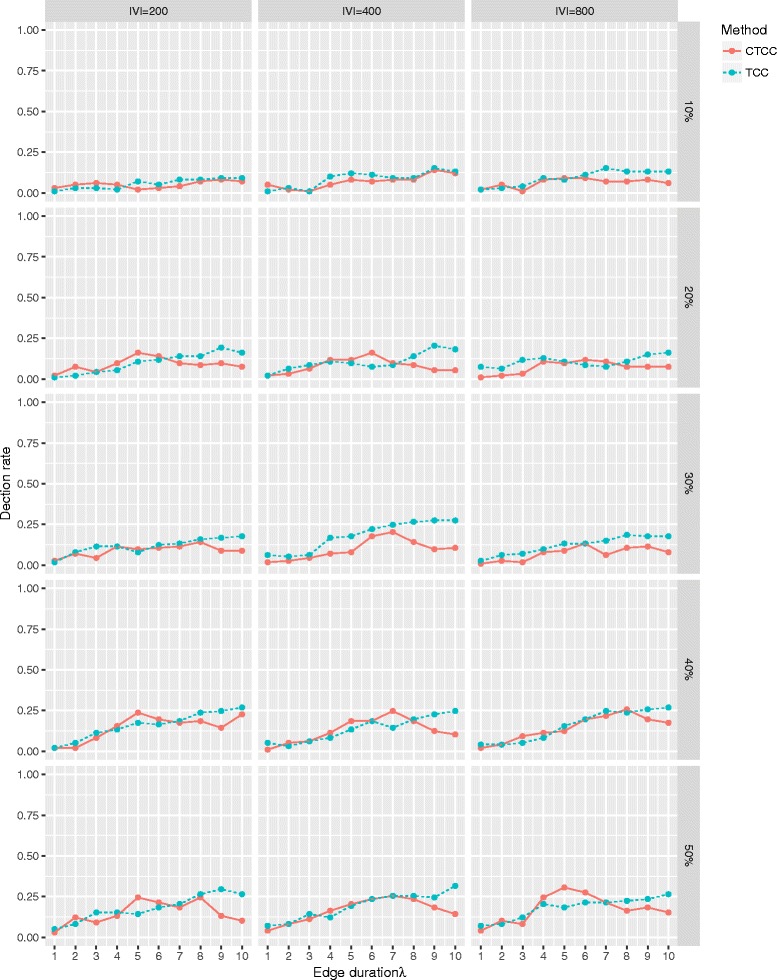
Impact of an excess of cloning on Spearman’s rank correlation coefficient *ρ*

Figure 10 shows clearly the expected problem of an excess of cloning in scenario *α*=100*%* (first row). As expected, the original TBC and no cloning (*n*_*c*_=0) are perfectly correlated (*ρ*=1.0), but the correlation of CTBC decreases with each additional clone. Note, that for *n*_*c*_=1 the length of the graph sequence is already doubled. However, the effect of an excess of cloning is less bad for longer edge durations *λ*.

The scenarios with lower observation rates show that the correlation values of CTBC are comparable to the values of TBC in settings with shorter edge durations or even larger for longer edge durations – despite the excess of cloning. Most important, although the performance of CTBC decreases with additional number of clones, it outperforms TBC even for large *n*_*c*_.

### Application to real dynamic networks

We used a real age-related dynamic network to investigate the performance of CTBC compared to TBC in a real world application. The dynamic network was created from a microarray human brain gene expression data set [18] that consists of 173 samples obtained from 55 individuals between 20 and 99 years of age. The reader may wish to refer to [11] for more details on the generation of this age-specific protein-protein-interaction network. From the original dynamic network, we selected only genes belonging to the KEGG metabolic pathways (hsa:01100) [29, 30] and their adjacent genes outside this pathway. This dynamic subnetwork contained 1,128 genes (vertices) and 31,643 temporal edges between 1,275 different vertex pairs which were connected by an edge at least in 1 out of 37 time points. Overall, the subnetwork contained 506 permanent edges that were present at all 37 snapshots, but also 1,931 temporal edges that existed only for one snapshot. Disregarding the permanent edges, the subnetwork showed a right skewed distribution of short to long edge durations.

To verify that the subnetwork kept the dynamic behavior of the whole network, we compared both regarding their dynamic edge density, that is the ratio between the observed number of edges at time *t* and the total number of possible edges at that time point. The dynamic edge density was similar for both networks the original network at all time points.

We used all observed 37 time points to calculate the *true* TBC of the dynamic subnetwork and ranked the vertices according to their TBC value. Then we selected every fourth snapshot to build an incomplete graph sequence with nine snapshots. The incomplete graph sequence contained 23% of the original 31,643 temporal edges that were present in 80% of the original 1,275 vertex pairs. Vertices were ranked according to their TBC and CTBC value estimated in the incomplete graph sequence. CTBC was calculated ten times where the number of clones *n*_*c*_ between snapshots was increased from one to ten.

The performance of TBC and CTBC was measured by the absolute rank difference (ARD) that compares the estimated ranks of the incomplete graph sequence to the true ranks of the complete graph sequence. Results are summarized in Table 1. It can be seen that all versions of CTBC$\phantom {\dot {i}\!}^{(n_{c})}, n_{c}=1,\ldots,10,$ outperformed TBC regarding the median, the first and third quartile as well as the interquartile range of the ARD. Further, the ARD of all CTBC versions showed smaller variability than of TBC. The median of CTBC has its minimum for seven or more clones, while the first and third quartiles are lowest for CTBC ^(4)^. The similarity of CTBC versions with six or more clones per snapshot and their coincident improvement of the ARD compared to TBC suggests that CTBC is robust against false positive edges introduced by cloning. We further calculated Spearman’s rank correlation coefficient *ρ* between the true ranks and the estimated ranks by TBC and CTBC. Albeit all methods achieved a high positive correlation with the true ranks (*ρ*≥0.89), CTBC had higher correlation values than TBC in all versions.

**Table 1:** TBC and CTBC performance regarding absolute rank differences to true ranks and Spearman’s *ρ*

Method	1st Qt.	Median	3rd Qt.	*ρ*
TBC	27.5	81.5	90.0	0.89
CTBC ^(1)^	24.0	49.5	65.0	**0** **.** **9** **3**
CTBC ^(2)^	19.0	45.5	55.0	**0** **.** **9** **3**
CTBC ^(3)^	16.5	36.5	50.0	**0** **.** **9** **3**
CTBC ^(4)^	**1** **5** **.** **0**	35.0	**4** **7** **.** **0**	**0** **.** **9** **3**
CTBC ^(5)^	17.0	35.0	48.0	0.92
CTBC ^(6)^	16.0	34.5	59.0	0.92
CTBC ^(7)^	17.0	**3** **4** **.** **0**	61.0	0.92
CTBC ^(8)^	16.0	**3** **4** **.** **0**	63.0	0.92
CTBC ^(9)^	18.0	**3** **4** **.** **0**	68.0	0.92
CTBC ^(10)^	18.0	**3** **4** **.** **0**	70.0	0.92

CTBC$\phantom {\dot {i}\!}^{n_{c}}$ was calculated using *n*_*c*_ clones per snapshots. Bold numbers indicate the minimum value

**Fig. 11:**
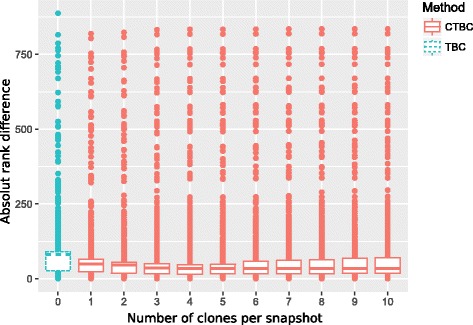
Results of the age-related dynamic brain network. Box plots of the absolute rank difference for the age-related dynamic brain network. The incomplete graph sequence with 9 snapshots was built on every 4th snapshot from the original graph sequence that consisted of 37 snapshots in total. The network included 1128 vertices. CTBC was calculated with different number of clones between snapshots. Very high absolute rank differences were caused by unobserved rare edges, that were crucial for the connectivity of (groups of) vertices in the dynamic network

Since incomplete graph sequences might completely miss some edges, the centrality values of vertices being incident to missing edges can be heavily biased. It is obvious that due to the information loss of these edges even cloning cannot decrease the ARD. In the real data example, this is reflected by very high absolute rank differences in all versions of CTBC and TBC, marked as outliers in the box plots (see Fig. 11).

## Discussion and conclusion

To the best of our knowledge this is the first work that introduced the problem of incomplete graph sequences when calculating temporal centrality measures. Our extension of existing temporal centrality measures addresses this problem by adding ‘clones’ of observed snapshots as extra snapshots into the graph sequence. The idea was motivated by real world dynamic networks, where edges occur for shorter and longer time durations rather than only during the specific observed snapshot. Furthermore, incomplete graph sequences are the rule rather than the exception in experimental and observational studies, where typically only a few snapshots of the total graph sequence can be obtained due to ethical, technical or financial reasons with varying time length between snapshots.

Since the clone temporal centralities augment the original graph sequence by adding snapshots, we needed an algorithm that can handle large graph sequences in reasonable time. With our new algorithm REN (Reversed Evolution Network) (shortest) temporal paths can be detected efficiently along a successively by one snapshot reduced graph sequence. The time complexity of the algorithm is linear in the number of snapshots and hence it allows the calculation of temporal centrality measures even in settings with long graph sequences.

Using the clone temporal betweenness centrality (CTBC) as an example for clone temporal centralities, our simulation studies demonstrate a superiority of CTBC relative to the original temporal betweenness centrality (TBC) [15] with respect to Spearman’s *ρ* and the detection rate of the most important network vertex. We also applied CTBC and TBC to a data set of an age-related gene expression network of the human brain, consisting of edges with shorter and longer durations. The analysis confirmed the better performance of CTBC compared to TBC. Both, the results from the simulation study and the real data example showed that the cloned temporal centralities are affected by an excess of cloning, since the true edge durations will tend to be overestimated, which again can result in the detection of false temporal paths. Except in data scenarios with short edge durations, cloning still provides better results even if too many clones were introduced in the observed snapshot sequence. There are three intuitive explanations why our approach outperforms the original approach even under an excess of cloning:

1. Not all wrongly introduced temporal paths due to cloning are *shortest* temporal paths and hence will not alter the cloned temporal centrality measures that are based on shortest temporal paths.
2. The original approach does not only miss true shortest temporal paths, it also *detects* false shortest temporal paths. This is due to the definition of a shortest temporal path: it is the temporal paths with the smallest number of hops and halts of all temporal paths between two vertices. For example, assume that there exist only two temporal paths, starting at a specific snapshot. Further, let one of them be a shortest temporal path. If only the longer temporal path can be found - due to the incomplete graph sequence - it will be falsely declared as a shortest temporal path.
3. If a shortest temporal path is missed, some of its subpaths as well as paths including this shortest temporal path will be missed too. Cloning snapshots raises the chance of finding at least some of those temporal paths.

However, while cloning snapshots is easy to implement, it cannot compensate for unobserved edges, resulting in inaccurate centrality values. Moreover, our method does not rely on probabilistic models describing the evolution of a dynamic network. Hence, we plan to investigate whether using probabilistic models for dynamic networks or exploiting a priori knowledge about the network topology can improve the estimation of temporal centrality measures.

Based on our results, we recommend using our clone temporal centrality measures in settings of incomplete graph sequences instead of the original temporal centrality measures. Additionally, using REN will improve computational speed in settings of long graph sequences. The R-code of our methods is available upon request from the authors and will be made available on CRAN.

## Additional file

Additional file 1 Clone temporal closeness centrality (CTCC). Definition of the clone temporal closeness centrality. (PDF 96 kb)

## References

[1] Holme P (2015). Modern temporal network theory: a colloquium. Eur Phys J B.

[2] Volz E, Meyers LA (2007). Susceptible–infected–recovered epidemics in dynamic contact networks. Proc R Soc London B: Biol Sci.

[3] Wölfer R, Faber NS, Hewstone M (2015). Social network analysis in the science of groups: cross-sectional and longitudinal applications for studying intra- and intergroup behavior. Group Dyn: Theory, Res Pract.

[4] Gao C, Liu J, Zhong N (2010). Network immunization and virus propagation in email networks: experimental evaluation and analysis. Knowl Inform Syst.

[5] Holme P, Saramäki J (2012). Temporal networks. Phys Rep.

[6] Hulovatyy Y, Chen H, Milenković T (2015). Exploring the structure and function of temporal networks with dynamic graphlets. Bioinformatics.

[7] Holme P, Saramäki J (2013). Graph Metrics for Temporal Networks.

[8] Tang J, Musolesi M, Mascolo C, Latora V, Nicosia V (2010). Analysing information flows and key mediators through temporal centrality metrics. Proceedings of the 3rd Workshop on Social Network Systems. SNS ’10.

[9] Kostakos V (2009). Temporal graphs. Phys A: Stat Mech Appl.

[10] Boccaletti S, Latora V, Moreno Y, Chavez M, Hwang DU (2006). Complex networks: Structure and dynamics. Phys Rep.

[11] Faisal FE, Milenković T (2014). Dynamic networks reveal key players in aging. Bioinformatics.

[12] Tang J, Scellato S, Musolesi M, Mascolo C, Latora V (2010). Small-world behavior in time-varying graphs. Phys Rev E.

[13] Grindrod P, Higham DJ, Parsons MC, Estrada E (2011). Communicability across evolving networks. Phys Rev E.

[14] Pan RK, Saramäki J (2011). Path lengths, correlations, and centrality in temporal networks. Phys Rev E.

[15] Kim H, Anderson R (2012). Temporal node centrality in complex networks. Phys Rev E.

[16] Alsayed A, Higham DJ (2015). Betweenness in time dependent networks. Chaos, Solitons Fractals.

[17] Katz L (1953). A new status index derived from sociometric analysis. Psychometrika.

[18] Berchtold NC, Cribbs DH, Coleman PD, Rogers J, Head E, Kim R, Beach T, Miller C, Troncoso J, Trojanowski JQ, Zielke HR, Cotman CW (2008). Gene expression changes in the course of normal brain aging are sexually dimorphic. Proc Nat Acad Sci.

[19] Blonder B, Wey TW, Dornhaus A, James R, Sih A (2012). Temporal dynamics and network analysis. Methods Ecol Evolu.

[20] Liang Q, Modiano E. Survivability in time-varying networks. In: 35th Annual IEEE International Conference on Computer Communications, INFOCOM 2016, San Francisco, CA, USA, April 10–14, 2016: 2016. p. 1–9.

[21] Li F, Chen S, Huang M, Yin Z, Zhang C, Wang Y (2015). Reliable topology design in time-evolving delay-tolerant networks with unreliable links. IEEE Trans Mobile Comput.

[22] Scellato S, Leontiadis I, Mascolo C, Basu P, Zafer M (2013). Evaluating temporal robustness of mobile networks. IEEE Trans Mobile Comput.

[23] Kempe D, Kleinberg J, Kumar A (2002). Connectivity and inference problems for temporal networks. J Comput Syst Sci.

[24] Berman KA (1996). Vulnerability of scheduled networks and a generalization of menger’s theorem. Networks.

[25] Costenbader E, Valente TW (2003). The stability of centrality measures when networks are sampled. Soc Netw.

[26] Borgatti SP, Carley KM, Krackhardt D (2006). On the robustness of centrality measures under conditions of imperfect data. Soc Netw.

[27] Magnien C, Tarissan F (2015). Time evolution of the importance of nodes in dynamic networks. Proceedings of the 2015 IEEE/ACM International Conference on Advances in Social Networks Analysis and Mining 2015. ASONAM ’15.

[28] Cormen TH, Leiserson CE, Rivest RL, Stein C (2009). Introduction to Algorithms.

[29] Kanehisa M, Sato Y, Kawashima M, Furumichi M, Tanabe M (2016). KEGG as a reference resource for gene and protein annotation. Nucleic Acids Res.

[30] Kanehisa M, Goto S (2000). KEGG: Kyoto encyclopedia of genes and genomes. Nucleic Acids Res.

