# Supplementary material for: Clone temporal centrality measures for incomplete sequences of graph snapshots
Source: BMC Bioinformatics. 2017 May 16;18:261. doi: 10.1186/s12859-017-1677-x (PMC5434573; doi:10.1186/s12859-017-1677-x)
Supplement: Additional file 1 — Clone temporal closeness centrality (CTCC). Definition of the clone temporal closeness centrality. (PDF 96 kb) [file 12859_2017_1677_MOESM1_ESM.pdf]

Clone temporal closeness centrality

In static network settings the closeness centrality of a vertex a describes how close a is to all other vertices $c \in V \backslash a$, in the network specified by the shortest paths. This centrality measure was extended [1, 2] for a graph sequence $\mathcal{G} = G_1, \ldots, G_S$ by proposing the *temporal closeness centrality* (TCC) for a vertex a as

$$TCC_{1,S}(a) = \sum_{k=1}^{S-1} \sum_{c \in V \backslash a} \frac{1}{|\gamma_{k,m,S}(a,c)|}, \tag{1}$$

where $|\gamma_{k,m,S}(a,c)|$ denotes the length of the shortest temporal path between vertices a to c over the snapshot sequence $G_k, \ldots, G_S]$. TCC captures the dynamics by summing over the graph sequence that gets successively reduced by one snapshot. In analogy to CTBC, we propose the *clone temporal closeness centrality* (CTCC) as an extension of Eq. (1):

$$CTCC_{1,S}(a) = \sum_{k=1}^{S} \sum_{j_k=1}^{J_k} \sum_{c \in V \backslash a} \frac{1}{|\gamma^{j_k}_{k,m,S}(a,c)|}, \tag{2}$$

where $|\gamma^{j_k}_{k,m,S}(a,c)|$ denotes the length of a shortest temporal path starting at the j_k-th clone of the k-th snapshot. If there is no such temporal path from a to c then the distance between a and c is defined as infinite and we assume that $\frac{1}{\infty} = 0$.

References

[1] Tang, J., Musolesi, M., Mascolo, C., Latora, V., Nicosia, V.: Analysing information flows and key mediators through temporal centrality metrics. In: Proceedings of the 3rd Workshop on Social Network Systems. SNS '10, pp. 3–136. ACM, New York, NY, USA (2010)

[2] Kim, H., Anderson, R.: Temporal node centrality in complex networks. Physical Review E **85**, 026107 (2012)
